# Misokinesia is a sensitivity to seeing others fidget that is prevalent in the general population

**DOI:** 10.1038/s41598-021-96430-4

**Published:** 2021-08-26

**Authors:** Sumeet M. Jaswal, Andreas K. F. De Bleser, Todd C. Handy

**Affiliations:** 1 Department of Psychology, University of British Columbia, 3406-2136 West Mall, Vancouver, BC V6T 1Z4 Canada; 2 Faculty of Psychology and Educational Sciences, Ghent University, Campus DunantDunantlaan 2, 9000 HenriGent, Belgium

**Keywords:** Psychology, Human behaviour

## Abstract

Misokinesia––or the ‘hatred of movements’––is a psychological phenomenon that is defined by a strong negative affective or emotional response to the sight of someone else’s small and repetitive movements, such as seeing someone fidget with a hand or foot. Among those who regularly experience misokinesia sensitivity, there is a growing grass-roots recognition of the challenges that it presents as evidenced by on-line support groups. Yet surprisingly, scientific research on the topic is lacking. This article is novel in systematically examining whether misokinesia sensitivity actually exists in the general population, and if so, whether there is individual variability in the intensity or extent of what sensitivities are reported. Across three studies that included 4100 participants, we confirmed the existence of misokinesia sensitivity in both student and non-student populations, with approximately one-third of our participants self-reporting some degree of sensitivity to seeing the repetitive, fidgeting behaviors of others as encountered in their daily lives. Moreover, individual variability in the range and intensity of sensitivities reported suggest that the negative social-affective impacts associated with misokinesia sensitivities may grow with age. Our findings thus confirm that a large segment of the general population may have a visual-social sensitivity that has received little formal recognition.

## Introduction

Misokinesia––or the ‘hatred of movements’––is a psychological phenomenon that is defined as a strong negative affective or emotional response to the sight of someone else’s small and repetitive movements, such as seeing someone mindlessly fidgeting with a hand or foot^1^. Among those who regularly experience misokinesia, there is a growing recognition of the challenges that it presents, as evidenced by blossoming on-line support groups. Yet surprisingly, scientific research on the topic is lacking. In fact, as late as July 15, 2021, a search for the term “misokinesia” on Web of Science (in all databases) returned no hits, either in the title of a paper or as a listed topic. Given this literal absence of scientific insight, the goal of our work presented here was to begin building an empirical foundation for understanding misokinesia and its potential social impacts.

If misokinesia has yet to be the topic of a scientific report, however, it does get an occasional mention in research articles. In particular, it receives passing recognition as a visual analog to *misophonia*^2^. In particular, misophonia (Greek *misos* = hatred, *phone* = sound) is defined as a disorder of decreased tolerance to specific sound^3^ and/or aversive emotional responses to human-produced sounds like chewing and lip-smacking^4^. The phenomenon, first identified by Johnson in the 1990s initially called “Selective Sound Sensitivity Syndrome”(4S)^5^, and was later coined “misophonia” by Jastreboff and Jastreboff^6^. Within the limited but expanding misophonia literature, one peer-reviewed study has in fact objectively reported on the prevalence of misokinesia, which was found to be 11.9% (or 5 patients) out of a 42 patient sample recruited from a hospital website for misophonia sufferers^1^. Nevertheless, the sample in this study was small, and it was restricted to a clinical population of individuals who were actively seeking support for their misophonia. What thus remains unknown is whether misokinesia sensitivity may be reliably reported in the general population (or populations not actively seeking treatment for misophonia concerns), and if so, whether there may be individual variability in the intensity or extent of what misokinesia sensitivities are reported.

As such, our aim in the set of studies reported here was to begin addressing these and related questions. Our approach involved three empirical steps. We first conducted an initial pilot study to assess whether misokinesia sensitivities would be reliably reported in a large sample of university undergraduates, based on a simple yes/no answer to a question asking about seeing fidgeting movements in others. Confirming many individuals do report such sensitivities, we then conducted an initial study again in a university undergraduate sample with three aims in mind: (1) to establish a basic prevalence rate, (2) to assess potential individual variability in reported impacts of misokinesia sensitivities, and (3) to determine whether misokinesia sensitivities may be associated with altered patterns of visual attentional performance. Finally, we then conducted a second study to assess prevalence rates and individual variability in misokinesia sensitivities in a more general, non-university population. In all three studies we included assessments of misophonia sensitivities in order to inform on the question of co-morbidity between misokinesia sensitivity and misophonia sensitivity. The end result is what we believe to be the first in-depth scientific exploration of what is a surprisingly common human phenomenon––a sensitivity to the visual presence of others who are fidgeting.

## Pilot study

The goal of our initial pilot study was to make an initial assessment of misokinesia sensitivity in a non-clinical undergraduate population in order to determine whether a more thorough investigation would be justified. A total of 2751 individuals (ages 17–66; Median = 20, SD = 3.27; 2028 female, 701 male, 3 trans-gender, 19 declined to identify) were recruited through the Human Subject Pool (HSP) on-line study recruitment portal for students enrolled in undergraduate courses in the Department of Psychology at the University of British Columbia (UBC). All participants provided informed consent prior to participation and were reimbursed 0.5 extra course credits. All protocols were approved by the UBC Behavioural Research Ethics Board, and all methods were performed in accordance with the relevant guidelines and regulations.

Our pilot study involved administering on an on-line questionnaire that asked two yes/no questions. The first question was used to assess the prevalence of misokinesia sensitivity in our sample: *Do you ever have strong negative feelings, thoughts, or physical reactions when seeing or viewing other peoples' fidgeting or repetitive movements (e.g., seeing someone's foot shaking, fingers tapping, or gum chewing)?* The second question was used to assess the prevalence of misophonia sensitivity in our sample: *Do you ever have strong negative feelings, thoughts, or physical reactions to specific or repetitive sounds, such as those from the mouth (e.g., hearing someone's eating, slurping, chewing, whispering, smacking, gum popping *etc*.) or other body parts (e.g., hearing someone's finger snapping, joint cracking, or foot tapping)?*

For the misokinesia sensitivity question, a total of 1053 students (or 38.3%) responded *yes*, while for the misophonia sensitivity question, a total of 1406 students (or 51.1%) responded *yes*. In terms of co-morbidity rates, a total of 872 students (or 31.7%) reported *yes* for both questions. In terms of misokinesia sensitivity rates within each sex, a total of 874 females (or 43.1%) and 173 males (or 24.7%) responded *yes* to the misokinesia sensitivity question. In terms of misophonia sensitivity rates within each sex, a total of 1118 females (or 55.1%) and 280 males (or 39.9%) responded *yes* to the misophonia sensitivity question.

Taken together, our findings suggest that misokinesia sensitivities do in fact extend to the general population (or populations not specifically seeking treatment for misophonia concerns), as more than one-third of our undergraduate sample reported experiencing some level of misokinesia sensitivity. Moreover, the numerically higher rate reported for females vs. males, and the reported level of co-morbidity with misophonia sensitivities parallel rates previously reported in a clinical population^1^, results which provide a measure of normative validity for our pair of assessment question. Given these initial confirmatory results, we then designed a study to not just confirm these initial prevalence rates, but to extend them in two critical ways––by examining individual variability in the strength and/or extent of reported misokinesia sensitivities in an undergraduate population, and by investigating whether these sensitivities may be associated with heightened visual-attentional sensitivities.

## Study 1

First, in terms of assessing individual variability in misokinesia sensitivities, presently there are no validated misokinesia assessment instruments. However, the Misophonia Assessment Question (MpAQ) was developed by Johnson and revised by Dozier^7^ to appraise the degree to which an individual experiences negative thoughts, feelings, and emotions regarding misophonic sounds. In Study 1, we thus adapted the MpAQ to ask about visual rather than auditory issues, thereby creating the Misokinesia Assessment Questionnaire (or MkAQ) to assess the degree to which an individual experiences negative thoughts, feelings, and emotions regarding misokinesic visual stimuli. In so doing, this had the additional benefit of allowing for a more direct comparison of misokinesia and misophonia sensitivities both within and between individuals.

Second, as a visual issue defined by a heightened salience for repetitive or fidgeting-based movements, we wanted to examine the possible cognitive correlates of misokinesia, or cognitive mechanisms that may contribute to the condition. In particular, could misokinesia be associated with either an increased inability to ignore distracting stimuli in the visual periphery, or an increased susceptibility of reflexively orienting visual attention to peripheral visual events? Given anecdotal reports of misokinesia as a subjectively experienced phenomenon (e.g., people commonly report a heightened attention to the fidgeting movements of others), either or both of these possibilities may be plausible. If so, it would suggest that misokinesia may be understood, at least in part, as an attention-related phenomenon.

Accordingly, Study 1 included two different behavioral assessments of visual attentional performance. One was a modified distractor interference paradigm, where participants performed a simple target detection task at fixation while ignoring brief kinetic-based distractors in the visual periphery; this was used to assess the ability of participants to inhibit peripheral attentional orienting. The other was a traditional reflexive attentional cuing paradigm, where participants performed a spatially cued target detection task in the visual periphery^8^; this was used to assess the magnitude of participants' peripheral attentional orienting. If misokinesia sensitivity is associated with altered visual attentional responsivity, it predicted that there should be a correlation between the degree of misokinesia sensitivity (as indexed by the MkAQ) and performance in these two behavioral attentional assessments. In other words, individuals that are more bothered by visual distractions in their daily lives were predicted to show evidence of greater distractor interference and/or stronger orienting responses to peripheral attentional cues, relative to those not reporting misokinesia sensitivities.

## Methods

### Participants

A total of 689 individuals were recruited through the UBC HSP on-line study recruitment portal; this number was based on our recruitment goal of running as many participants as possible during the fall, 2019 semester at UBC. Data from 39 of these participants were excluded for either leaving the MkAQ or MpAQ incomplete, or not completing the behavioural task, leaving a final sample of 650 individuals (514 females, 124 males, 2 non-binary, 1 agender, 9 declined to answer; the age range was 18–44, with 594 between the ages of 18–24, 28 between the ages of 25–34, 4 between the ages of 35–44, and 24 who declined to answer). All participants provided informed consent prior to participation, self-reported as free of neurological problems, including no reports of head injuries resulting in loss of consciousness for over 5 min, or stroke, meningitis, and/or seizures, were fluent in English and had normal range vision (with or without corrective lenses), and received 0.5 extra course credits. Participants completed the tasks in a quiet room with only the researcher present in order to control for unwanted or environmental conditions (i.e., potential auditory or visual distractors). All protocols were approved by the UBC Behavioural Research Ethics Board, and all methods were performed in accordance with the relevant guidelines and regulations.

### Procedures

After arriving at the laboratory and giving informed consent, each participant performed two behavioral tasks, as described below. Following completion of these tasks, they then filled out a set of questionnaires and were debriefed on our study. Total testing time took approximately 0.5 h.

#### Questionnaires

Participants filled out four online questionnaires in total through Qualtrics: A basic demographics form, the MkAQ (see Supplementary Fig. S1 online), the MpAQ^4^, and the State and Trait Anxiety Inventory^9^; however, the latter was collected for use in a different study and thus an analysis of those findings are not included below.

#### Behavioural tasks

Participants completed a visual detection task that required manual button-press responses. The behavioural tasks were completed on a computer running Psychopy 3 V3.2.4^10^ which displayed targets created using the GratingStim function^11^. Two different attentional assessments were included in Study 1––a distractor interference task, and a reflexive attentional cuing task. All 650 participants completed both, with the distractor task always performed first. However, as described below, we employed two different versions of the attentional cuing tasks, one that used a kinetic-based peripheral visual cue and the other that used a more canonical “flash” as a visual cue^8^. We included this manipulation of cue type to examine whether misokinesia sensitivities may be associated with a particular sensitivity to kinetic-based visual events; cue-type was manipulated between-subjects, with participants run in the first half of the semester performing the “kinetic” version of the attentional cuing task, and participants run in the second half of the semester performing the more traditional "flash" version of the attentional cuing task. For all behavioral tasks, at the beginning of each session, the participants performed practice trials and were given the opportunity to ask the experimenter any clarifying questions. Accuracy and speed of response were emphasized equally to the subjects. The viewing distance for all participants was kept at 57 cm from the centre of the computer screen; actual stimulus sizes and display locations are reported below for each of the behavioural tasks.

##### Distractor interference task

**Figure 1:**
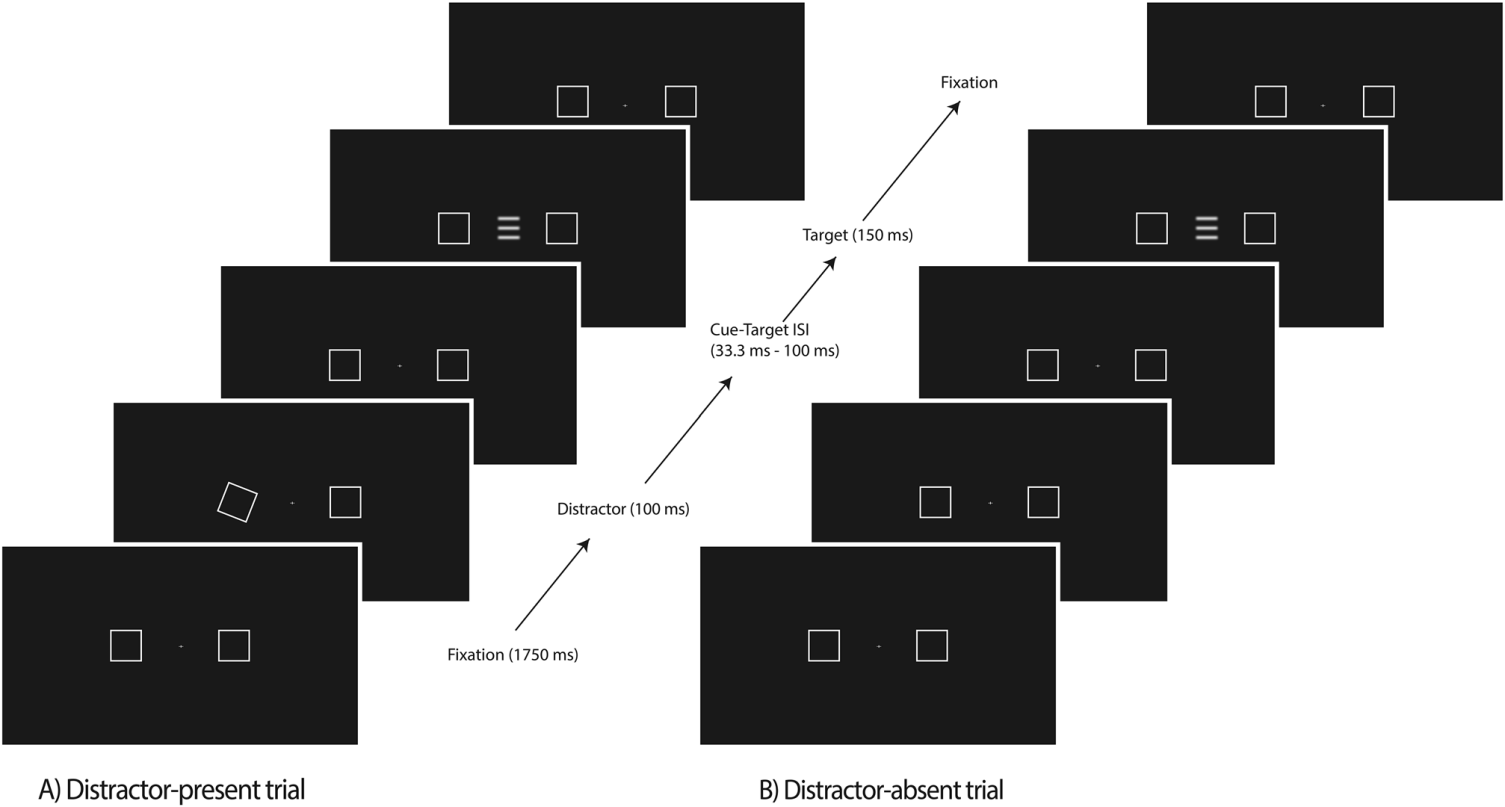
Sequence and timing of stimulus events in the distractor interference task of Study 1. Trial-sequence for the (**A**) distractor-present at the left location, and the (**B**) distractor-absent is shown. ISI = interstimulus interval. Participants were able to input their response at the final fixation panel. Max response time allowed = 1000 ms.

This was a target detection task that required making speeded button presses whenever a target stimulus was presented at fixation; trial sequences and timings are shown in Fig. 1.

Two boxes, one to the left and one to the right of the fixation cross, remained on-screen throughout the trial block. These peripheral boxes were demarcated by the outlines of 4.28° square boxes and were located 7.68° to the centre fixation cross (0.74°). The target stimulus consisted of three 0.7° horizontal sine wave grating lines inside a square box of 2.81°, and was presented at the fixation cross; participants responded to the target onset with a button-press (spacebar) using their right index finger when the target stimulus appeared. On *distractor-present* trials (66.6% of trials), one of the peripheral boxes was briefly “wiggled” just prior to the onset of the target (i.e., the orientation of the box was briefly rotated by 15° clockwise and then back to its original orientation, as a kinetic-based visual distraction). On *distractor-absent* trials (33.3% of trials), the target was presented without a preceding box “wiggle.” To reduce anticipatory target responses, targets were only presented on half of the trails, for each trial type. Participants completed four blocks of 36 trials per block (24 distractor-present, 12 distractor-absent), with the distractor-present trials equally split (but randomly varying) between a “wiggle” of the left vs. right boxes.

##### Attentional cuing task

**Figure 2:**
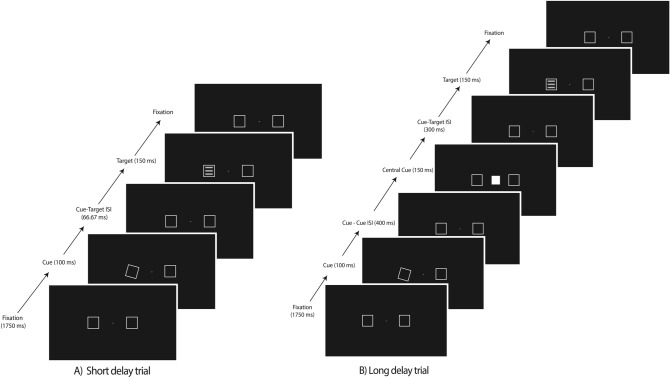
Sequence and timing of stimulus events in the attentional cuing task (kinetic cue) in Study. Trial-sequence for the (**A**) validly-cued at the left location for the short cue-target delay, and the (**B**) validly-cued at the left location for the long cue-target delay is shown. ISI = interstimulus interval. Participants were able to input their response at the final fixation panel. Max response time allowed = 1000 ms.

**Figure 3:**
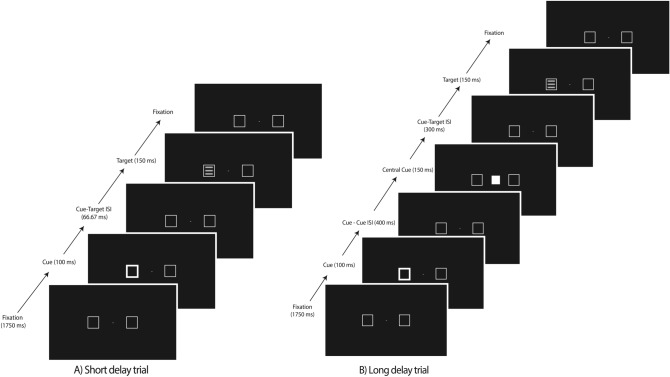
Sequence and timing of stimulus events in the attentional cuing task (flash cue) in Study 1. Trial-sequence for the (**A**) validly-cued at the left location for the short cue-target delay, and the (**B**) validly-cued at the left location for the long cue-target delay is shown. ISI = interstimulus interval. Participants were able to input their response at the final fixation panel. Max response time allowed = 1000 ms.

This was a target detection task that required making speeded button presses whenever a target stimulus was presented at a peripheral location either to the left or right of fixation; trial sequences and timings were adapted from Handy et al.^12^ and are shown in Figs. 2 and 3. Two boxes, one to the left and one to the right of the fixation cross, remained on-screen throughout the trial block. Participants responded with a button-press (spacebar) using their right index finger when the target stimulus appeared on the screen. In the short-delay condition, the target was presented following a peripheral cue. In the long-delay condition, the peripheral cue was followed by a second (central) cue presented at fixation see^12^. In the version of the task that used a kinetic-based attentional cue, we employed the same box “wiggle” as described above for the distractor interference task (Fig. 2); in the version of the task that used a more canonical “flash” as an attentional cue, the outline of one of the boxes was briefly thickened (Fig. 3).

The central cue was a 0.33° filled white square. On *validly-cued* trials, the target appeared inside the cued peripheral box. On *invalidly-cued* trials, the target appeared inside the uncued peripheral box (or the box on the opposite side of fixation from the cued box). To reduce anticipatory responses, we also included *catch* trials, where a peripheral and central cue were presented, but without a subsequent target. Participants completed four blocks of 36 trials per block (12 valid, 12 invalid trials, 12 catch trials), with the cued trials equally split (but randomly varying) between short and long cue-target delays.

## Results

Our analyses focused on three a priori issues of interest––confirming a basic prevalence rate for misokinesia sensitivities in our undergraduate sample, establishing a distribution of individual variability in the strength or magnitude of misokinesia sensitivity within our sample, and establishing whether misokinesia sensitivity is associated with altered visual attentional performance, relative to those not reporting misokinesia sensitivities.

### Prevalence and variability

Our assessment of prevalence was based on the MkAQ. Mirroring analysis of the MpAQ^4^, the MkAQ asks 21 different questions concerning misokinesia-related issues, with each question being answered using a rating scale of 0 to 3 to indicate severity/intensity, with a 0 indicating the issue is experienced “none of the time” and a 3 indicating the issue is experienced “almost all the time.” Given this coding, summing an individual's responses gives an index of misokinesia severity, in that a higher sum––or “sum score”––indicates a greater number of issues experienced and/or a higher severity/intensity of issues. To facilitate comparison of the prevalence rates of misokinesia sensitivity and misophonia sensitivity between Study 1 and our pilot study (which based prevalence on a binary-choice question), we then divided participants into two groups based on their MkAQ and MpAQ sum scores––those with a sum score of 0 or 1 (or reporting no/minimal misokinesia/misophonia sensitivity), and those with a sum score of 2 or more (or reporting non-minimal misokinesia/misophonia sensitivity).

In terms of misokinesia sensitivity rates, a total of 392 students (or 60.3%) reported a sum score of 2 or more on the MkAQ, while in terms of misophonia sensitivity rates, a total of 460 students (or 70.8%) reported a sum score of 2 or more on the MpAQ. In terms of co-morbidity rates, a total of 246 students (or 37.8%) reported a sum score of 2 or more on both questionnaires. In terms of misokinesia sensitivity rates within each sex, a total of 320 females (or 62.3%) and 62 males (or 50.0%) reported a sum score of 2 or more on the MkAQ. In terms of misophonia sensitivity rates within each sex, a total of 366 females (or 71.2%) and 83 males (or 66.9%) reported a sum score of 2 or more on the MpAQ.

**Figure 4:**
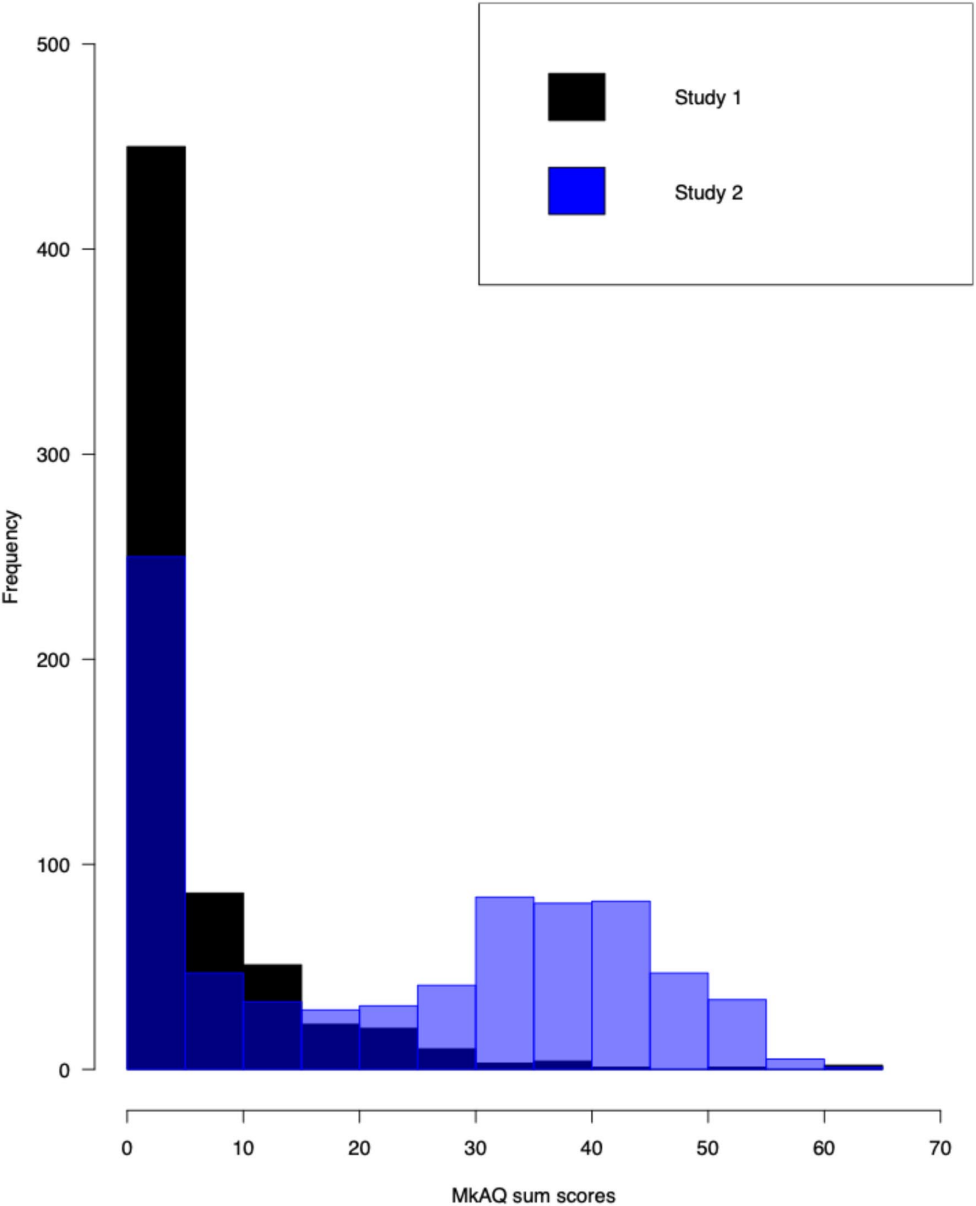
Frequencies of MkAQ sum scores plotted for Study 1 (black) and Study 2 (blue).

To assess individual variability in the strength or magnitude of misokinesia sensitivities, we first plotted the MkAQ sum scores as a frequency histogram (Fig. 4). As can be seen, scores were positively skewed, with a majority of participants reporting a sum score of 5 or less. More specifically, 258 participants (or 39.7%) had a sum score of 0 or 1 (or what was defined above as no/minimal misokinesia sensitivity), 192 participants (or 29.5%) had a sum score of 2–5, and 200 participants (or 30.8%) had a sum score of 6 or higher; these groupings we then labeled as “no misokinesia sensitivity” (or noM), “low misokinesia sensitivity” (or lowM), and “high misokinesia sensitivity” (or hiM) for subsequent analyses. For females, 194 (or 37.7%) classified as noM, 156 (or 30.4%) classified as lowM, and 164 (or 31.9%) classified as hiM. For males, 62 (or 50.0%) classified as noM, 31 (or 25.0%) classified as lowM, and 31 (or 25.0%) classified as hiM.

### Attentional performance

Our goal in analyzing the performance data was to examine whether attentional performance systematically varies with misokinesia sensitivity. Because the range of MkAQ sum scores was so positively skewed (Fig. 4), rather than use a correlational approach to performance analyses, we treated misokinesia sensitivity as a between-group factor based on the classification above––noM, lowM, and hiM––and interrogated the data using repeated-measures analyses of variance (ANOVAs).

#### Distractor interference task

All 650 participants completed the distractor interference task. Mean reaction times (RT) and accuracy data (*d'* and beta) are presented in Table 1 as a function of trial type (distractor-present, distractor-absent) and group (noM, lowM, and hiM). Participants appeared to be faster and more accurate in responding on distractor-present vs. distractor absent trials, an effect that did not seem to vary as a function of misokinesia group. We confirmed this pattern via a mixed model ANOVA with within-subject factor of trial type and between-subject factor of group. Although we found a significant main effect of distractor for RT (*F* (1, 647) = 1195.42, *p* < 0.001, ${\eta }_{p}^{2}$ = 0.65) and *d’* (*F* (1, 647) = 203.11, *p* < 0.001, ${\eta }_{p}^{2}$ = 0.24), we failed to show any main effect of group or a group x trial type interaction for either RT or *d’* analyses (both *p* ≥ 0.27). We had *post-hoc* power of 5% for observing our null between groups effect (${\eta }^{2}$ = 0.001).

**Table 1:** Mean reaction time, d’ and beta across subjects for the distractor interference task in Study 1, as a function of cue condition and MkAQ scores (noM = a sum score of 0 or 1 lowM = a sum score of 2–5, hiM = a sum score of 6 or higher).

	N	Cue condition	Measure		
			reaction time (ms)	*d′*	Beta
noM	258	Distractor-present	243 (41)	3.984 (0.401)	1.348 (0.906)
		Distractor-absent	282 (48)	3.736 (0.251)	2.053 (0.732)
lowM	192	Distractor-present	244 (45)	3.980 (0.427)	1.256 (0.729)
		Distractor-absent	283 (53)	3.725 (0.260)	2.023 (0.791)
hiM	200	Distractor-present	244 (51)	3.926 (0.664)	1.457 (1.059)
		Distractor-absent	279 (58)	3.691(0.415)	2.168 (1.001)

Total participants (N) in each group. Mean values, with standard deviations in parentheses.

#### Attentional cuing task––kinetic cue

A subset of 191 participants were run in this task; data from 23 participants were excluded for not completing the questionnaires, the behavioural task, or having a high number of false alarms (3 or more in either the short- or long-delay condition), resulting in a final sample of 168 participants (*N* = 139 females, 22 males, 1 gender, 6 declined to respond; ages 18–34.) Mean RTs are presented in Table 2 as a function of trial type (validly-cued vs. invalidly-cued), cue-target delay (long or short), and group (noM, lowM, and hiM). It appeared that responses were faster on invalidly-cued trials compared to validly-cued trials, and also during long cue-target delay relative to short cue-target delay trials. Both of these effects, however, did not seem to vary as a function of misokinesia sensitivity groups. We confirmed this data pattern via a mixed model analyses of variance for RT with within-subject factors of trial type and cue-target delay, and between-subject factor of group. We found a significant main effect of cue (*F* (1,165) = 319.08, *p* < 0.001, ${\eta }_{p}^{2}$ = 0.66), cue-target delay (*F* (1,165) = 91.79, *p* < 0.001, ${\eta }_{p}^{2}$ = 0.36), and a significant interaction of cue and cue-target delay conditions (*F* (1,165) = 188.92, *p* < 0.001, ${\eta }_{p}^{2}$ = 0.53). However, we failed to show any group differences (*F* (2,165) = 1.36, *p* = 0.26). We had *post-hoc* power of 5% for observing our null between groups effect (${\eta }^{2}$ = 0.016).

**Table 2:** Mean reaction time across subjects for the attentional cuing task (kinetic cue) in Study 1, as a function of cue condition, cue-target delay, and MkAQ scores (noM = a sum score of 0 or 1 lowM = a sum score of 2–5, hiM = a sum score of 6 or higher).

	N	Cue condition	
		Validly-cued RT (ms)	Invalidly-cued RT (ms)
**Short cue-target delay**			
noM	66	327 (55)	322 (50)
lowM	52	313 (49)	309 (46)
hiM	50	315 (51)	309 (50)
**Long cue-target delay**			
noM	66	322 (48)	276 (44)
lowM	52	311 (49)	267 (41)
hiM	50	311 (51)	266 (51)

Total participants (N) in each group. Mean values, with standard deviations in parentheses.

#### Attentional cuing task––flash cue

A subset of 498 participants were run in this task; data from 38 participants were excluded for leaving the questionnaires incomplete, not finishing the behavioural task, or having a high number of false alarms (3 + in either the short- or long-delay condition), resulting in a final sample of 460 participants (*N* = 356 females, 100 males, 1 non-binary, 3 declined to respond; ages 18–44). Mean RTs are presented in Table 3 as a function of trial type (validly-cued vs. invalidly-cued), cue-target delay (long or short), and group (noM, lowM, and hiM). Responses appeared to be faster in on invalidly-cued trials compared to validly-cued trials, and also during long cue-target delay relative to short cue-target delay trials. However, response patterns did not appear to vary as a function of group. We confirmed this data pattern via a mixed model ANOVA with within-subject factors of trial type and cue-target delay, and between-subject factor of group. We found significant main effect of cue (*F* (1,457) = 757.59, *p* < 0.001, ${\eta }_{p}^{2}$ = 0.62), cue-target delay (*F* (1,457) = 216.95, *p* < 0.001, ${\eta }_{p}^{2}$ = 0.32), and a significant interaction of cue and cue-target delay conditions (*F* 1,457) = 694.61, *p* < 0.001, ${\eta }_{p}^{2}$ = 0.60). However, we failed to show any group differences (*F* 2,457) = 0.25, *p* = 0.78). We had *post-hoc* power of 5% for observing our null between groups effect (${\eta }^{2}$ = 0.001).

**Table 3:** Mean reaction time across subjects for the attentional cuing task (flash cue) in Study 1, as a function of cue condition, cue-target delay, and MkAQ scores (noM = a sum score of 0 or 1 lowM = a sum score of 2–5, hiM = a sum score of 6 or higher).

	N	Cue condition	
		Validly-cued RT (ms)	Invalidly-cued RT (ms)
**Short cue-target delay**			
noM	188	318 (50)	316 (48)
lowM	131	317 (60)	321 (57)
hiM	141	320 (67)	322 (65)
**Long cue-target delay**			
noM	188	317 (52)	267 (50)
lowM	131	321 (55)	275 (58)
hiM	141	322 (67)	275 (65)

Total participants (N) in each group. Mean values, with standard deviations in parentheses.

## Discussion

Our goals in Study 1 were three-fold. First, we wanted to confirm the general prevalence rate for misokinesia in a second student-aged sample. In that regard, we found that almost one-third of our participants had a sum score of 6 or more on the MkAQ, a rate not inconsistent with the 38.3% of participants from our pilot study who reported *yes* to the question of whether they had visual sensitivities to fidgeting and like behaviors. While the MkAQ measure of prevalence does not readily translate into a binary choice measure as used in our pilot study, together this pair of findings provide empirical support for the conclusion that misokinesia sensitivities are indeed present––if not widespread––in non-clinically defined populations.

Second, we wanted to perform an initial assessment of individual variability in self-reported misokinesia sensitivities within a non-clinical population. In examining the frequency distribution of MkAQ sum scores as shown in Fig. 4, our data indicate that there is clear variability in the extent to which sensitivities are reported and thus presumably experienced. While in the participants having a sum score of 2 or more there is a strong positive skew in the frequency distribution (i.e., the majority had sum scores of 15 or less), there were in fact a number of individuals reporting much more extensive issues and sensitivities. This suggests that misokinesia sensitivity is not necessarily a binary phenomenon in terms of symptomology, but rather, the impacts experienced by individuals can widely differ in breadth and/or intensity.

Finally, from a cognitive perspective, we wanted to determine whether misokinesia sensitivity could be associated with either an increased inability to ignore distracting stimuli in the visual periphery, and/or an increased susceptibility of reflexively orienting visual attention to peripheral visual events. However, in all three behavioural tasks we found no evidence to support either possibility. In our distractor interference paradigm, target responses were actually faster and more accurate on distractor-present trials across all three groups, relative to distractor absent trials. This suggests that far from distracting attention away from the target's location at fixation, the distractor served as a reliable temporal warning cue as to the target's pending presentation. Likewise, in both versions of the reflexive attentional orienting paradigm, there were again no between-group differences observed. While overall attentional cuing effects were absent at the short cue-target delay (i.e., we did not show behavioral evidence of increased attention at the cued location in the visual periphery), target responses at the long cue-target delay were significantly faster at the uncued (vs. cued) peripheral location, an effect consistent with inhibition of return (or IOR;^8^). IOR is normative at long cue-target delays in reflexive orienting paradigms e.g.,^12^, suggesting that both our orienting tasks were in fact influencing reflexive visual attentional mechanisms at least to some degree. More importantly though, there were again no significant between-group differences observed. As such, while it may remain to further probe potential attentional correlates of misokinesia sensitivity, our initial evidence is consistent with the conclusion that reflexive visual attentional mechanisms may not make substantive contributions to misokinesia sensitivity.

## Study 2

While our findings from Study 1 suggest that misokinesia sensitivity is not associated with altered patterns of attentional performance, we did confirm the original results from our pilot study demonstrating that it is prevalent in the general population––approximately 1 in 3 participants in our two studies reported some level of sensitivity. Further, we found that for those experiencing misokinesia sensitivities, there is a high degree of individual variability in how those sensitivities are manifest or impact their daily lives. Given these conclusions regarding prevalence, we wanted to conduct a second study with the explicit goal of expanding our assessment of misokinesia prevalence in the general population, and in particular, expanding it beyond a relatively young, student-aged sample. More specifically, our aims in this final study were two-fold. First, we wanted to determine whether our estimate of an approximately 33% prevalence rate for misokinesia would hold in an older, more demographically diverse sample, and second, we wanted to examine whether individual variability in this sample would show a similar frequency distribution in reported misokinesia sensitivities as to what we found in Study 1 in our student-age sample.

### Methods

We recruited 1007 adults from Amazon Mechanical Turk (MTurk) for the current study; 242 of these participants failed a set of three separate attention checks included in our study protocol and were not included in analyses (see Supplementary Table S1 online). This resulted in a final sample of 765 participants (N = 244 females, 516 males, 3 non-binary, 2 trans gender; age range = 18–93, median = 32 years). Participants were reimbursed $1.50 USD for their participation. The study was approved by the Behavioural Research Ethics Board at the University of British Columbia, and all methods were performed in accordance with the relevant guidelines and regulations. All participants provided informed consent prior to participation. In terms of procedures, we replicated two aspects of our earlier studies: (1) we asked participants the two questions described above in our pilot study, to assess whether they had problems related to their misokinesia sensitivity and/or misophonia sensitivity, and (2) participants completed the four questionnaires (demographics, MkAQ, MpAQ, State and Trait Anxiety Inventory) described above in Study 2; again, the anxiety measures were used for a separate study.

### Results

For our misokinesia question, a total of 275 participants (or 35.9%) responded *yes*, while for our misophonia question, a total of 325 participants (or 42.5%) responded *yes*. In terms of co-morbidity rates, a total of 195 participants (or 25.5%) reported *yes* for both questions. In terms of misokinesia rates within each sex, a total of 93 females (or 38.1%) and 182 males (or 35.3%) responded *yes* to the misokinesia question, while a total of 169 females (or 69.3%) and 395 males (or 76.6%) reported a sum score of 2 or more on the MkAQ. In terms of misophonia sensitivity rates within each sex, a total of 120 females (or 49.2%) and 204 males (or 39.5%) responded *yes* to the misophonia question, while a total of 188 females (or 77.1%) and 397 males (or 76.9%) reported a sum score of 2 or more on the MpAQ.

To assess individual variability in the strength or magnitude of misokinesia sensitivities, as per Study 1 we first plotted the MkAQ sum scores as a frequency histogram (Fig. 4) and subdivided participants into three groups––noM, lowM, and hiM. As can be seen in Fig. 4, the frequency distribution of MkAQ sum scores showed a somewhat bimodal distribution. More specifically, 197 participants (or 25.8%) had a sum score of 0 or 1 (noM), 53 participants (or 6.9%) had a sum score of 2–5 (lowM), and 515 participants (or 67.3%) had a sum score of 6 or higher (hiM). This frequency distribution appeared to differ from the distribution obtained in Study 1 (Fig. 4), an observation that was confirmed via a two-sample Kolmogorov–Smirnov test (D(650,765) = 0.476, *p* < 0.001) using critical values generated via the Real Statistics Resource Pack^13^.

## Discussion

We had two goals in Study 2. First, we wanted to assess the prevalence rate for misokinesia sensitivities in a non-student, non-clinically-defined population, and we found that approximately one-third (35.9%) of the respondents reported experiencing some level of misokinesia sensitivity in their lives. This percentage is consistent with the prevalence rate found in our pilot study that also used a binary-choice question for assessing misokinesia prevalence. If we apply the same measure of prevalence rate as in Study 1––the percentage of participants having a sum score of 6 or more––the prevalence rate nearly doubles (67.3%). While we discuss these differences between measures in our general discussion below, the more central point remains that misokinesia sensitivities were indeed found to be prevalent in a non-student-based sample from the general population.

Second, we wanted to examine the distribution of misokinesia sensitivities in a non-student, non-clinically defined sample. In that regard, we found that there was a significant difference between the frequency distributions from Studies 1 and 2, with the latter showing a more bimodal pattern and higher percentage of participants falling in the hiM category, relative to the former. Why might this be? Demographically, the MTurk-based sample in Study 2 was older than our student-aged sample from Study 1, and contained a higher percentage of male participants. There were also clear differences in the distribution of ethnicities between the two participant samples, as can be seen in Table 4. Below we discuss how these demographic factors may help to explain the observed differences in frequency distributions of misokinesia sensitivities.

**Table 4:** Ethnicity breakdown for Study 1 and Study 2 participants.

	Study 1		Study 2	
	N	%	N	%
Aboriginal or First Nations	1	0.15	25	3.27
African	8	1.23	82	10.72
East Asian	279	42.92	16	2.09
European (Caucasian)	143	22.00	378	49.41
Hispanic	11	1.69	103	13.46
Middle Eastern	28	4.31	–	–
Multi-ethnic	30	4.62	31	4.05
Pacific Islander	1	0.15	–	–
Prefer not to answer	22	3.38	–	–
South Asian	74	11.38	110	14.38
Southeast Asian	53	8.15	20	2.61
Total	650		765	

Total number (N) and percentage (%) of each ethnicity that participated in Study 1 and Study 2.

## General discussion

Our findings reported here represent what to the best of our knowledge is the first systematic examination of misokinesia sensitivity and its prevalence in the general population. Across three studies that collectively sampled over 4100 individuals, we found that approximately one-third self-reported some degree of misokinesia sensitivity to the repetitive, fidgeting behaviors of others as encountered in their daily lives. These results support the conclusion that misokinesia sensitivity is not a phenomenon restricted to clinical populations, but rather, is a basic and here-to-fore under-recognized social challenge shared by many in the wider, general population. But beyond this immediate conclusion, our set of studies inform on several further questions that help to begin building a deeper scientific understanding of this visual-social sensitivity.

First, is misokinesia sensitivity always co-morbid with misophonia sensitivity? Our findings suggest not. While co-morbidity rates exceeded 25% in each of our three population samples, we also consistently found a percentage of individuals reporting misokinesia sensitivities in the absence of any misophonia sensitivities. And consistent with the original prevalence report of Schröder et al.^1^ in a small clinical population, we also consistently found a percentage of individuals reporting misophonia sensitivity but not misokinesia sensitivities. Taken together, this pattern of co-occurrence between the two phenomena suggests that while they are often experienced together in an individual, misokinesia itself is not simply a co-morbidity or visual analog of their misophonia sensitivity; for some individuals the challenge of seeing others fidget is experienced in the absence of any corresponding auditory-social correlates.

Second, to what extent might misokinesia sensitivities vary across individuals? In this regard, we found that there was indeed clear variability in the strength and/or extent of misokinesia sensitivities in the populations we sampled in Studies 1 and 2. When participants were subdivided into groups based on MkAQ scores, we noted that in Study 1 approximately one-third of the participants were categorized as having no misokinesia sensitivity, one-third had low sensitivity, and one-third had high sensitivity. In contrast, when we subdivided the participants from Study 2 using the same trichotomous groupings, there was an apparent shift in the pattern of variability: one-fourth of the participants in this population were categorized as having no sensitivities, 7% were categorized as having low sensitivity, and the largest subgroup—approximately two-thirds of the participants—were categorized as having a high level of misokinesia sensitivity. In other words, a larger proportion of participants in Study 2 demonstrated high misokinesia sensitivities, relative to the participants in Study 1. One possibility is that this difference in variability between our two studies could simply reflect an issue of sampling noise. However, given that the sample in Study 2 had an older mean age, and significantly higher percentages of males and Caucasians relative to Study 1, another possibility for future study is that the intensity of how misokinesia is experienced may in fact vary with core demographic factors such as age, sex, and/or ethnicity. Regardless though, our data indicate that misokinesia sensitivity is not experienced as a binary phenomenon, but rather, there is in fact wide variability in the range of sensitivities individuals experience.

Finally, towards understanding the underlying basis for misokinesia sensitivity, might it be associated with heightened visual-attentional sensitivities? Despite the well-powered behavioral experiments we ran in Study 1, our findings showed no systematic support for this possibility. In particular, we found that misokinesia sensitivities were not associated with either an increased inability to ignore distracting events in the visual periphery, nor an increased susceptibility of reflexively orienting visual attention to sudden events in the visual periphery. While it is always critical to interpret such null results with care, they do begin to help frame basic cognitive questions about the phenomenon. For instance, misokinesia sensitivity could in fact be associated with altered visual-attentional function, but either (1) the paradigms used in our study were not valid assessments of these attentional correlates, or (2) individuals with misokinesia sensitivities may be well-practiced at controlling visual attention in a top-down manner as a compensatory strategy for mitigating their discomfort, strategies that could mask attentional correlates of the condition. On the other hand, given that misophonia has been strongly associated with altered affective reactivity to trigger sounds^14^, it could also be the case that misokinesia sensitivity does not involve altered attentional functioning, and instead, it too may be more tied to heightened affective reactivity to visual triggers. These now become important questions to begin pursuing if we are to build a neurocognitive understanding of the phenomenon.

### Limitations

In closing, there are also a number of important limitations to consider regarding our studies and their findings. The first concerns the actual questions we used to assess misokinesia sensitivity. In our initial Pilot Study we used a simple yes/no question to divide our sample into two groups––those with versus without some level of misokinesia sensitivity. Our intention in so doing was not to definitively inform on misokinesia or misophonia prevalence. Rather, it was to make an initial, coarse assessment of misokinesia sensitivity using a deliberately liberal criteria in order to determine if a more in-depth investigation might be warranted. That we found a larger proportion of our undergraduate participants reported a misophonia sensitivity in our Pilot Study (51.1%) relative to what has been reported in previous clinically-oriented studies of misophonia using more stringent criteria, such as Wu et al.^14^ (20%), Zhou et al.^15^ (6%), and Naylor et al.^16^ (37%) is thus not surprising. As such, while we believe our Pilot Study findings are informative and important to report for thorough documenting of our investigation, we stress that this initial assessment was never designed to provide a definitive prevalence rate for either misokinesia or misophonia in non-clinically defined populations.

Likewise, for Studies 1 and 2 we had no validated misokinesia measure or questionnaire we could rely on in order to make a more fine-grained assessment of misokinesia sensitivities. As such, we chose to adapt the unvalidated MpAQ to develop MkAQ because it was both a straight-forward process to translate the auditory-focused MpAQ to the visual-focused MkAQ, and using a visual analog to the MpAQ would facilitate a direct comparison of misokinesia and misophonia sensitivities within the same individual. Nevertheless, the MkAQ emphasized social/clinical impacts rather than providing a clear accounting of the more immediate, subjective effects of misokinesia sensitivity. More specifically, the questions in the MpAQ––on which the MkAQ was based––focus on the emotional impacts of misophonia and address possible social problems that can arise for those who experience the phenomenon. As the MpAQ was designed based on clinical interviews conducted by an audiologist^17^, it is possible that merely adapting the questionnaire for studying misokinesia sensitivity did not capture actual misokinesia symptoms as they occur when in the presence of a visual trigger. The MkAQ, thus, does not measure subjective experiences of triggers, responses, or coping mechanisms, and this could again help to explain our null attentional performance results. Namely, if misokinesia sensitivity is predicated on visual-emotional symptoms when dealing with an actual trigger stimulus rather than the social-emotional impacts of having to manage possible exposures to trigger stimuli (as captured by the MkAQ), it could reveal possible between-group differences in attentional performance that are obscured when grouping is based on social-emotional impacts as per Study 1. Taken all together, this highlights the need to consider refining and validating measures of both misokinesia and misophonia sensitivities as a logical next research step.

Second, it is also important to note that the key stimulus used in our attentional paradigm—the kinetic movement of the peripheral boxes—may not have been an effective distractor/cue, for individuals with misokinesia sensitivity. That is, the kinetic movement of the peripheral boxes may not have been a valid proxy for human fidgeting, or the kinds of stimuli that are visual triggers in misokinesia sensitivity. This possibility is certainly consistent with what is known about the nature of processing within visual cortex. The so-called ventral visual stream that underpins visual object processing bifurcates into areas that respond to animate vs. inanimate objects e.g.,^18^, and numerous neuroimaging studies have confirmed that while ventrolateral visual brain regions are activated by animate objects, it is more ventromedial regions that are activated by inanimate objects. This functional dissociation raises the question of whether misokinesia sensitivity could be specifically associated with altered attentional sensitivity to either animate objects in general, or perhaps even more selectively, to human movements exclusively. For example, if our mirror neuron systems are activated when seeing the behaviors of others^23^, this would explain not just why misokinesia sensitivities could be exclusive to human movement, but given that fidgeting is associated with psychomotor agitation and anxiety^24–26^, it could also explain the aversive responses to seeing such movements in others, as it may induce these negative states in the observer. These now become important open questions to explore.

Third, we note that the prevalence rate for misophonia sensitivity that we report in Study 1 is potentially high in comparison to prior studies of misophonia sensitivity in undergraduate populations. We suggest that one critical factor may be the demographic profile of the undergraduate samples between studies. For example, in Wu et al.^14^ only ~ 4% of their undergraduate sample was classified as Asian, whereas in our sample we had > 50% east Asian and south Asian participants. However, a second critical factor would be the actual criteria used for assessing misophonia sensitivity, as our use (and cutoffs) of the MAQ may not have good convergence with approaches used for assessment in other studies, which is another critical limitation. Nevertheless, we again note that the primary goal of our study was not to study misophonia sensitivity, but rather, to establish that misokinesia sensitivity is a common experience that appears to be prevalent in the general population. That our findings raise vital questions for future research, such as whether social-perceptual sensitivities may vary with individual factors such as race, ethnicity, cultural background, gender, and sex, speaks to the importance of continuing such work.

### Conclusion

Despite the open questions and limitations outlined above, our data firmly establish that misokinesia sensitivity is indeed prevalent in the more general (non-clinically-defined) population, and that many people may be experiencing negative social-emotional impacts from something that has received little formal recognition. Moreover, as our findings suggest, the scope and intensity of these negative social-affective impacts may in fact increase with age. Yet while our findings highlight a number of pressing questions to address going forward, the end result here is that we have confirmed something long under-appreciated about the human condition––we don't just frequently fidget as a species, but as well, many of us are challenged by being in the visual presence of others who are doing so.

## Supplementary Information

Supplementary Information 1.
