# Supplementary material for: Misokinesia is a sensitivity to seeing others fidget that is prevalent in the general population
Source: Sci Rep. 2021 Aug 26;11:17204. doi: 10.1038/s41598-021-96430-4 (PMC8390668; doi:10.1038/s41598-021-96430-4)
Supplement: Supplementary file 1 — Supplementary Information 1. [file 41598_2021_96430_MOESM1_ESM.pdf]

Supplementary Methods

Misokinesia is a Sensitivity to Seeing Others Fidget That is Prevalent in the General Population

Sumeet M. Jaswal, Andreas K. F. De Bleser, & Todd C. Handy

Supplementary Methods

RATING SCALE:<br>0 = not at all, 1 = a little of the time, 2 = a good deal of the time, 3 = almost all the time	0	1	2	3
1. My visual issues currently make me unhappy	○	○	○	○
2. My visual issues currently create problems for me.	○	○	○	○
3. My visual issues have recently made me feel angry.	○	○	○	○
4. I feel that no one understands my problems with certain visuals.	○	○	○	○
5. My visual issues do not seem to have a known cause.	○	○	○	○
6. My visual issues currently make me feel helpless.	○	○	○	○
7. My visual issues currently interfere with my social life.	○	○	○	○
8. My visual issues currently make me feel isolated.	○	○	○	○
9. My visual issues have recently created problems for me in groups.	○	○	○	○
10. My visual issues negatively affect my work/school life (currently or recently).	○	○	○	○
11. My visual issues currently make me feel frustrated.	○	○	○	○
12. My visual issues currently impact my entire life negatively.	○	○	○	○
13. My visual issues have recently made me feel guilty.	○	○	○	○
14. My visual issues are classified as 'crazy'.	○	○	○	○
15. I feel that no one can help me with my visual issues.	○	○	○	○
16. My visual issues currently make me feel hopeless.	○	○	○	○
17. I feel that my visual issues will only get worse with time.	○	○	○	○
18. My visual issues currently impact my family relationships.	○	○	○	○
19. My visual issues have recently affected my ability to be with other people.	○	○	○	○
20. My visual issues have not been recognized as legitimate.	○	○	○	○
21. I am worried that my whole life will be affected by visual issues.	○	○	○	○

Supplementary Figure S1. Misokinesia Assessment Questionnaire (MkAQ)

Supplementary Methods

Supplementary Table S1. The three attention checks in study 2.

1. Research in attention shows that people, when answering questions, prefer not to pay attention and minimize their effort as much as possible. Some studies show that over 50% of people don't carefully read questions. If you are reading this question and have read all the other questions, please select the box marked 'other' and type 'attention' in the box below. Do not select "Coronavirus" Thank you for participating and taking the time to read through the questions carefully!

   What was this study about?

   - Daily life activities Anxiety
   - Coronavirus
   - Other (please specify) ______________

1. Correct response: other (please specify) attention

2. During the MkAQ, participants were provided the following prompt: *Select the option 'a little of the time'* from the following options:

0 = NOT AT ALL	1 = A LITTLE OF THE TIME	2 = A GOOD DEAL OF THE TIME	3 = ALMOST ALL THE TIME

2. Correct response: a little of the time

3. During the MpAQ, participants were provided the following prompt: *Select the option 'a good deal of the time'* from the following options:

0 = NOT AT ALL	1 = A LITTLE OF THE TIME	2 = A GOOD DEAL OF THE TIME	3 = ALMOST ALL THE TIME

3. Correct response: a good deal of the time
